# ‘Until You Get the Diagnosis You're Forever in Limbo’—Parents' Experiences of Waiting for an Attention‐Deficit/Hyperactivity Disorder Assessment With Child and Adolescent Mental Health Services

**DOI:** 10.1111/hex.70569

**Published:** 2026-01-28

**Authors:** Ellen Hedstrom, Katarzyna Kostyrka‐Allchorne, Claire Ballard, Naomi James, Hannah Wright, David Daley, Cris Glazebrook, Jana Kreppner, Claire Cattel, Douglas Gordon, Natalie Gordon, Tessa Tuttlebee, Edmund Sonuga‐Barke

**Affiliations:** ^1^ Centre for Innovation in Mental Health, School of Psychology, Faculty of Environmental and Life Sciences University of Southampton Southampton UK; ^2^ Department of Psychology, School of Biological and Behavioural Sciences Queen Mary University of London London UK; ^3^ Division of Care for Long Term Conditions Florence Nightingale Faculty of Nursing, Midwifery & Palliative Care, King's College London London UK; ^4^ Hampshire and Isle of Wight Healthcare NHS Foundation Trust Southampton UK; ^5^ Academic Unit of Mental Health & Clinical Neurosciences, Faculty of Medicine &Health Sciences University of Nottingham Nottingham UK; ^6^ Department of Psychology, School of Social Sciences Nottingham Trent University Nottingham UK; ^7^ Independent Patient and Public Partner, King's College London London UK; ^8^ Department of Child and Adolescent Psychiatry Institute of Psychiatry, Psychology & Neuroscience, King's College London London UK; ^9^ Department of Child & Adolescent Psychiatry Aarhus University Arhus Denmark; ^10^ Department of Psychology The University of Hong Kong Pok Fu Lam Hong Kong

**Keywords:** ADHD, CAMHS, child mental health, clinical services, waitlist

## Abstract

**Background:**

Parents in the United Kingdom seeking an assessment for attention‐deficit/hyperactivity disorder (ADHD) for their child experience a significant wait before receiving an appointment with Child and Adolescent Mental Health Services (CAMHS), yet little has been written on how parents experience this period. Through qualitative interviews, we sought to understand how the period of waiting from being accepted onto a service waitlist and receiving a diagnostic assessment impacts parents and their children.

**Method:**

The study was nested within a large randomised controlled trial. We conducted semi‐structured interviews with 41 parents of children aged 5–11 years. 30% of parents had waited between 18 and 24 months on a CAMHS waitlist, with 10% waiting more than 2 years. Reflexive thematic analysis was used to analyse data.

**Results:**

At the point of the interview, around 50% of children were still waiting for an initial assessment. Six themes reflecting parents' uncertainty around the assessment process, lack of communication from services, the importance of receiving a diagnosis, difficulty accessing support and the negative impact of waiting on mental health and education, as well as recommendations to improve communication between services and families, emerged.

**Conclusion:**

Parents recognised the pressures on services to offer timely support; however, their well‐being could be substantially improved by more clarity around wait times, as well as more effective signposting and support from services concerning the assessment process. This may help alleviate some of the stressors associated with their child's assessment journey, such as feeling responsible for their child's difficulties and the burden of supporting their educational needs.

**Patient and Public Contribution:**

This study was nested within the OPTIMA trial, where PPI panel members provided ongoing support in various aspects of the study, including advising on participant communication, study design and data analysis. All PPI members have lived experience of having a neurodivergent child. For this study, the PPI co‐produced the interview schedule and took part in transcript analysis using a thematic framework approach. To acknowledge their contributions, members of the PPI panel are included as co‐authors.

## 1 Introduction

Attention‐deficit/hyperactivity disorder (ADHD) is characterised by age‐inappropriate and impairing levels of inattention, hyperactivity or impulsivity or their combination [1] with a worldwide prevalence of around 5% [2]. According to National Health Service (NHS) England, it is estimated that around 621,000 children aged 0–17 have ADHD [3]. Children with suspected ADHD are typically referred for diagnosis and treatment to Child and Adolescent Mental Health Services (CAMHS) via schools or General Practitioners (GPs). If the referral is accepted, after initial triage, the child is placed on a waitlist before being offered an appointment for an assessment. Several appointments may take place from the initial assessment until a diagnosis is established.

An independent study in 2023 by an ADHD charity found that the range of wait times in the United Kingdom varied from 5 weeks to 5 years. Furthermore, they found only 15% of Health Care Boards in England knew their waitlist times for children awaiting an ADHD assessment [4]. Recent data from NHS England shows that at the end of September 2025, 63.6% of children aged 0–17 who were waiting for an ADHD assessment had been waiting over a year, with around 35% of this group waiting for more than 2 years [3]. While the National Institute for Health and Care Excellence (NICE) guidelines do not explicitly advise a maximum time frame for ADHD assessments to commence [5], there is concern that long wait times may exacerbate ADHD symptoms and be detrimental to the mental health of the parent and child and the more general well‐being of the family [6, 7].

Long wait times may negatively impact families, leading to reduced engagement with services or dropping out of treatment [8]. Parents may also look for support from other agencies, which can be costly both in terms of time spent seeking support, but also in terms of the emotional and financial burden this can impose on parents [9]. While less research has focussed on the real impact of awaiting a diagnosis on children's well‐being, lengthy wait times have been found to impact education [10]. Furthermore, longitudinal studies have established the long‐term outcomes of children with ADHD and effects on mental health and education [11] and missed school days related to social and emotional problems [12].

New ways to manage the referral process are being trialled [13, 14]. For example, Valentine et al. found that psychoeducation, parental support and coaching showed improved clinical outcomes for young people waiting on a mental health service list, including autism spectrum disorder, self‐harm and anxiety/depression [14]. However, in a survey on waitlist interventions (WLIs), Valentine et al. found that a third of the trusts contacted did not offer WLI; furthermore, lack of staff needed to facilitate the interventions was a barrier to implementation [15].

Understanding how long wait times impact parents and children can help provide much‐needed support to families while they wait. In addition, understanding the needs of families can inform policy and the development of tools that not only support families but also reduce the burden on mental health services.

### 1.1 Study Aim

The key aim of this study was to understand how parents experience the time between their child's referral for an ADHD assessment being accepted by CAMHS and receiving a diagnostic outcome, and the impact this period had on them and their child. The study emerged from parental feedback at the end of the OPTIMA study, where parents shared their concerns about the support they had received from CAMHS during the wait, with a heavy focus on how they felt CAMHS had communicated with them. In conjunction with the PPI panel, an interview schedule was created focussing on three main research questions: How do parents experience communication and support from CAMHS while waiting for an assessment/diagnosis? How does the way in which CAMHS communicate with parents impact them and their child? What changes would parents like to see from CAMHS to improve the overall experience?

## 2 Materials and Methods

### 2.1 Design

The interviews were nested within the OPTIMA trial. Ethical approval for the trial was obtained from the Health Research Authority (REC reference number: 21/NW/0319) and funded by the National Institute for Health Research. OPTIMA participants were invited to take part in interviews on their experiences of being on a CAMHS waitlist. Participation was optional. Interviews took place between November 2023 and June 2024.

### 2.2 Participants

Purposive sampling ensured representation of parents from all three OPTIMA trial centres (London, Southampton and Nottingham) and inclusion of participants from diverse socio‐economic and ethnic backgrounds. Parents at different stages of the clinical pathway were included. Parents were invited via email and received a copy of the participant information sheet as well as a flyer with a simple explanation of the interview process, including example questions. All parents provided consent before taking part.

A total of 41 parents took part in the interviews. Their demographic data were representative of the overall OPTIMA sample (*n* = 352): 75.6% of parents identified as white, 91.5% as female and 68.3% were married or in a long‐term relationship. When asked about their child's diagnostic status, 48.8% were still waiting for the initial assessment (see Table 1).

**Table 1:** Participant demographics for interview participants.

Variable	*****N*** (%)**
Parent gender	
Male	2 (2.8)
Female	39 (97.2)
Parent ethnicity	
Black/Black British—Caribbean, African, other	4 (9.8)
Mixed race—White and Black/Black British	2 (4.9)
Mixed race—other	3 (7.3)
Chinese/Chinese British	1 (2.4)
Middle Eastern/Middle Eastern British—Arab, Turkish, other	1 (2.4)
White	30 (73.2)
Relationship status	
Single	13 (31.7)
Married, or in a long‐term relationship	28 (68.3)
Highest level of education	
No formal qualifications	1 (2.4)
Completed GCSE/CSE/O‐levels or equivalent (finished school aged 16)	5 (12.2)
Completed post‐16 vocational course	1 (2.4)
A‐levels/BTEC or equivalent (finished school aged 18)	7 (17.1)
Undergraduate degree or professional qualification	12 (29.3)
Postgraduate degree	15 (36.6)
Total household income	
Less than £16,000 a year	9 (21.9)
£16,000–£29,999 a year	7 (17.1)
£30,000–£59,999 a year	11 (26.8)
£60,000 or more a year	11 (26.8)
Prefer not to say	3 (7.3)
Current employment status	
Employed full‐time	12 (29.3)
Employed part‐time	13 (31.7)
Student	1 (2.4)
Homemaker	4 (9.6)
Self‐employed	7 (17.1)
Unable to work	4 (9.6)
Diagnosis status	
Complete ADHD diagnosis given	15 (36.6)
Complete ASD diagnosis given	2 (4.9)
Complete no diagnosis given	1 (2.4)
Diagnosis in process	3 (7.3)
Still waiting	20 (48.8)
Wait times	
7–12 months	3 (7.3)
12–18 months	10 (24.4)
18–24 months	12 (29.3)
24 months plus	4 (9.8)
Don't know/can't remember	12 (29.3)

### 2.3 Interviews

The interview schedule was co‐designed in conjunction with the OPTIMA Public and Patient Involvement Panel (PPI; see Appendix A). Three researchers, all of whom worked on the OPTIMA trial, conducted the interviews via MS Teams or on the phone, depending on parental preferences. Interviews lasted between 30 and 45 min. Parents were asked about their child's assessment journey since referral. The interview ended with a mood repair, where interviewees were asked about a positive topic. Participants were also offered a ‘safety' card that had been used throughout the trial, offering contacts for support organisations and crisis support numbers. Interview recordings (video and audio recordings) were stored securely on the KCL SharePoint. At the point of transcription, all personal data were redacted, including names (parent and children), location and any other identifying information. All recordings were deleted at the end of the study and only anonymised transcripts retained. Interviewees were given a unique ID number at the point of interview, which was stored securely on the university ShareDrive and password protected. Once all transcripts were complete, each ID number was assigned a pseudonym, ensuring confidentiality and anonymity [16] as well as giving participants voices an identity [17]. On completion, parents were given a £20 Amazon voucher as a thank you for their time.

### 2.4 Analytical Approach

Reflexive thematic analysis was employed [18]. Braun and Clarke's model allows for flexibility in its approach to meaning generation and the evolution of research questions [19]. With less‐researched topics, it also allows for data‐driven, inductive analysis. A team of four researchers transcribed and analysed the transcripts (three of whom had conducted the interviews). This ensured data familiarisation, the first step in thematic analysis [18]. NVivo versions 14/15 were used to generate initial codes and identify patterns to categorise data (Step 2). At this stage, four members of the PPI panel individually reviewed two transcripts with the coding from the research team to either support or challenge the initial interpretations. The research team then started searching for and reviewing themes (Steps 3 and 4). This was done through regular meetings within the research team to discuss the addition, removal or merging of codes, giving definitions to each code and eventually collating them into themes. In Step 5, the research team and PPI members met to discuss the research team's findings, refining and naming the themes. In the final step (6), a rich set of quotes from participants collated throughout the analytical process was used to illustrate the themes.

The team took a reflexive approach to analysis, recognising their personal and professional experiences, such as being a parent (E.H. and C.B.) or having personal experience of interacting with clinical services. Furthermore, the lived experience of the PPI members and how this shaped data interpretation was acknowledged. Parents' names were pseudonymised, which allows for data to be de‐identified without de‐personalising it [17].

## 3 Result

**Figure 1:**
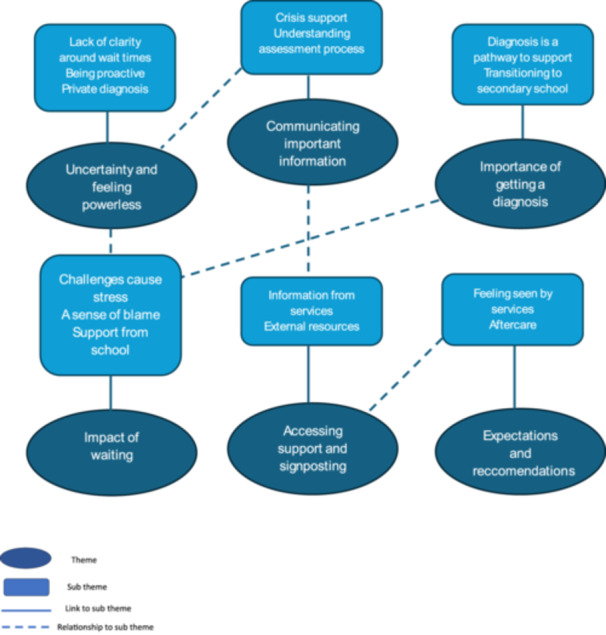
Thematic map. Adapted from [20].

Six themes emerged, each with several sub‐themes, allowing a deeper exploration of theme meanings [18] (see Figure 1).

### 3.1 Theme 1—Uncertainty Leads to Feeling Powerless

The first theme related to the period prior to the diagnostic assessment, which many parents described as a time of great uncertainty, arising from not knowing how long they would be waiting and what they could do to support their child during this time.

#### 3.1.1 Lack of Clarity Around Wait Times and Support

A key message from parents was that there was a lack of clarity about how long they would have to wait. While parents appreciated that the waitlists were dynamic and not necessarily based on a *first‐come, first‐seen system*, parents felt that transparency from services regarding estimated wait times was important because it enabled them to manage expectations as well as make plans for their family.

If they could tell me that next Christmas you're gonna have the thing, then at least I would know and try and plan—it's just not knowing, it's not helpful…

Jen

I mean be honest, they need to be honest and just say look, the wait is still 18 to 24 months. At least people know where they are

Fiona

Almost unanimously, parents felt that communication concerning their waitlist status was unsatisfactory, with only two parents stating that they had received confirmation that they were on the waitlist. Most parents were given little to no information about likely wait times. Moreover, this lack of communication meant some parents did not know that their child was still on the waitlist, causing added anxiety.

I was reassured that she is still on the waitlist, but I've heard nothing since, so I don't know if she is or not.

Trish

#### 3.1.2 The Importance of Being Proactive

The sub‐theme of being proactive encompassed parents' ability to follow up with services in terms of seeking information and support (rather than seeking help and support elsewhere). Being proactive allowed parents to regain some sense of agency. One parent commented that contact with the service made them feel ‘*like things are progressing rather than a big, black hole’ (Farah)*.

Parents also reflected on their own privilege of being able to advocate for their child and chase services for information and support.

I just think communication is key, right?… It was only through my personality where I tried to take control [in contacting the services].

Pete

#### 3.1.3 Implications of Seeking Alternative Help and Support

Some parents considered seeking a private assessment as another way in which they could regain some control over the assessment and diagnosis process. However, uncertainties surrounding the CAMHS waitlist, whether a private diagnosis would be officially recognised (e.g., by their child's school), as well as financial matters, made the decision to seek alternative care difficult for parents.

We are struggling a bit and it would be nice to know whether we should be trying to save up to get him seen privately, if it's gonna be years and years, then that's what we'd do. If it's gonna be another 6 months, then we'll wait.

Sarah

If I could guarantee that if I went private and that it would be accepted and that everything would be fine, then potentially I would find the money to go private.

Fiona

### 3.2 Theme 2—Communicating Important Information

#### 3.2.1 A Need for Crisis Support

Parents were asked if they knew what to do if they felt their child needed urgent support. This included knowledge on who to contact if their child's mental health deteriorated or if the child had expressed suicidal ideation, for example. For parents who had required *crisis care* for their child while waiting for their assessment and diagnosis, there were mixed responses. Some parents had struggled to find a contact number, while others had managed to contact a crisis team but had felt that the support was inadequate.

I did try and find the crisis mental health services or something like that and I just couldn't find the right place.

Hannah

I did call that number for my older one when he kept saying he was going to kill himself when he was very stressed, and they kind of just went “oh, you know, if he's autistic, that's how he expresses himself.”

Chloe

#### 3.2.2 Understanding the Assessment Process

Parents had varied experiences with services when it came to communication on the assessment process, which included pre‐assessment information such as how the assessments were structured, as well as understanding the process from assessment to diagnosis. Parents felt it was especially important to prepare their child for each meeting.

I would love to know before the initial appointment, what questions are you going to ask my child and how are you going to do it? I was quite anxious. What do I need to do?

Farrah

Also, as one parent found, information would be helpful to avoid disappointment in thinking the assessment period would be completed quicker than it actually took.

…you realise during the assessment, “oh, no, it's not the full one, it's just the initial assessment.” So that was a disappointment.

Farrah

Even when parents had received a date for their initial assessment, they were not always clear about what would happen during the assessment. This lack of clarity was seen as an additional stressor for both them and their child. Conversely, other parents felt that the post‐referral processes were explained clearly and that they knew what would be happening. This illustrates the inconsistency of the quality of services within and across the NHS Trusts.

…we're getting information from an individual who gave us direct information on what's going to happen, sort of pre‐talked to us, talked to our child, then talked to us afterwards as well. So it's a really clear bit of process.

Jon

### 3.3 Theme 3—The Importance of Getting a Diagnosis

#### 3.3.1 A Diagnosis Is the Pathway to Support and Understanding

Parents viewed getting a diagnosis as essential for accessing support and treatment for their child.

It's hard because there is no support, at the moment, until you get that diagnosis and you're forever in limbo.

Jayne

Without a diagnosis, some parents felt unable to request additional support from the school, and others believed that their family members (e.g., grandparents) viewed them as ‘bad’ parents, as they lacked a professional validation of their concerns and explanation of their child's behaviour.

They kind of brushed me off and said, basically I'm allowing him, they basically said he was spoiled and that was why [child] behaved the way he behaved. Which I was quite upset about.

Kat

#### 3.3.2 Transitioning Into Secondary School

Waiting for an assessment was especially stressful for parents with children transitioning into secondary school, who had concerns about whether the required support would be offered without an official diagnosis.

She's off to secondary school in September. And I feel like she's got no support. I don't know if she's ready. I wanted her to get help before this point, you know?

Gwen

Another parent had pre‐empted this concern by pursuing a private assessment, which she felt had benefited her child.

I felt really bad for [child] … technically if we hadn't got her done privately, we'd still be waiting and she's coming up to needing all those decisions about around like secondary school.

Trish

### 3.4 Theme 4—Accessing Support and Signposting

The fourth theme explored how parents experienced support and signposting, which included resources offered by CAMHS, as well as those sought through other sources such as social media and local groups. Many parents felt that they lacked information from services and knowledge on how to support their child while waiting for an assessment.

#### 3.4.1 From Services

This generally consisted of signposting to other organisations or parenting groups set up to offer support. There was a disparity both within and between local services regarding the type of support offered. For example, some parents were offered evidence‐based parent training programmes, such as the *New Forest Parenting Program* (NFPP) [21] or *Incredible Years* [22]. Other parents received a pack with information and signposting to support organisations in the local area.

When asked about what types of support from services would be useful, parents mentioned mental health support (for both parent and child) and strategies to help them better manage their child's behaviour.

Strategies that could have very easily been shared in some sort of PDF or videos online that … things we could have put in place earlier and without a diagnosis.

Jaz

One parent wanted information on the Right to Choose (RTC) process, which they felt might speed up the assessment process. RTC allows patients in some areas of the United Kingdom to ask their GP to refer them to any qualified provider registered with the NHS. For some, this could mean moving to a clinic with a shorter waitlist.

#### 3.4.2 Support Sought From External Resources

For most parents, online resources were the main source of support and information. This took the form of social media groups such as Facebook or parenting forums. Parents also found local support groups and charities helpful.

I joined a couple of Facebook groups and follow Instagram people who had ADHD, because a lot of what they were saying resonated with way the way [child] was … and then I would try them with her and it made sense. I did find that really supportive because … it made me feel less alone.

Jaz

There was wide variation in how parents used external sources of information; some read or watched everything they came across, while others were more selective, appreciating that there is a lot of misinformation about ADHD, especially on social media. Some parents described information and support from external sources as having a positive impact on them during the waiting period, both in terms of providing useful information as well as giving them emotional support and kinship.

My other half especially found it really useful to kind of have that (social media), partly with knowing what's going on with the process and other people's experience of that process but also come up with strategies….

Jon

Other parents felt that social media may prime parents to fear the worst outcome for their child, which prevented them from seeking information and support from informal online sources.

I didn't want to research too much into ADHD and then convince myself from what do you know, like look up stuff…. And even online, they scaremonger about the waiting list.

Charlie

One parent said that groups were ‘*mainly people who want to vent or get things off their chest’* while another parent found them *‘judgemental’*. This also led some parents to avoid informal sources of information.

And I'm quite a worrier. I have quite a low anxiety threshold and knowing me I'd read something I'll be like, Oh my God. So I don't.

Emma

### 3.5 Theme 5—The Impact of Waiting

Parents reported that the psychological impact of waiting was profound. It created additional parental stress and led to mental health difficulties for both parents and the referred children. Parents also referred to the negative impact that the delay in a diagnosis had on how their child was perceived at school.

#### 3.5.1 Practical Challenges Increase Stress

For parents, administrative tasks related to the process of getting their child assessed created additional stress, which, together with experiencing their child's distress, negatively affected their mental health.

And it's not just dealing with the child. It's like, it's the meetings. It's the appointments.

Michelle

Phrases like ‘*living a nightmare’* and ‘*resentful’* were used. Many parents had themselves struggled with their own mental health during the waiting process, and they described how much waiting had impacted them, which had led one parent to seek help for their own mental health problems.

I did go to my doctor and I got put on fluoxetine for anxiety. It's made me really anxious of everything: going out, like uhm how people are looking at me, and when they're not looking at me. And how I might how I might be viewed if I do have to restrain her and stuff.

Trish

#### 3.5.2 Feeling a Sense of Blame

Parents also recognised the impact that being on the waitlist had on their children. For some children, a lack of formal diagnosis contributed to feelings of blame or self‐doubt.

The frustrating thing was by that point she was already low self‐esteem, depression, anxiety. All these things because she just felt different and didn't know why. There was a point she was saying things like “I'm going to jump out the window”, “things would be better off without me here”.

Trish

#### 3.5.3 Lack of Understanding From School

For children struggling with attendance, school also became a challenge during the waiting period. Parents talked about difficulties with getting schools to listen to them and support their child.

I'm having a lot of issues with school at the moment. They're saying that because she's not fully diagnosed, I don't need to be talking to her about it. They actually got social services on me because they said that I was, what's the word? Erm, not making it up, but I think they were thinking I was kind of making it worse or something.

Michelle

we've wasted over 2 years of her education, it is a huge percentage. And in that time, she was just getting further and further behind.

Jaz

### 3.6 Theme 6—Expectations and Recommendations

The final theme concerned parents' expectations of the ADHD referral service and recommendations to improve families' experiences of being on a waitlist. For some parents, there was a clear disparity between what support they felt they needed and the support they received. However, other parents were realistic about their expectations. They recognised that clinical services were under great strain, which led to long wait times. Those parents wanted to make as few demands as possible, recognising that it was not the staff's fault that communication was lacking and appointments were delayed.

### 3.7 Feeling Seen by the Service With Regular Check‐Ins

Parents valued information on wait times while recognising that, due to systemic pressures and the dynamic nature of the waitlists, it could be hard to get even an approximate date for their appointment. Many parents felt forgotten and even worried that their child was no longer on the waitlist. Feeling seen by services was deemed important to parents' well‐being.

They don't ever just phone or email just to say, “Oh, you've been on here a while. Like, how are things going? Is there anything else we can do to support you in the process?”' (Michelle). This was echoed by one parent, Evie ‘I think maybe just a bit of a touching base every few months’.

One parent suggested that having a named key worker would have been valuable, so they knew who to contact when they needed help. Another parent suggested a digitalised system whereby parents could log on to check their status (and be reassured that they were still active on the waitlist). This could also offer links to support organisations.

### 3.8 Aftercare and Treatment

Many parents received very little aftercare from services once their child had received a diagnosis, which was contrary to their expectations. Some parents expressed that they had assumed things would improve for their child post‐diagnosis, including face‐to‐face support for both parent and child and strategies to support child behaviour. However, this expectation was often very different from reality.

I think the assessment itself is very good. It's the afterwards that that is just completely empty of anything and any support whatsoever … there was no follow up on what we should do next so it just feels like they dropped a lot of information on us, which was the diagnosis, and then left us basically to deal with it on our own.

Rae

One father, Pete, was clear on the information that would have been helpful, which included holistic support encompassing the wider family.

A transparent, clear process. An assessment report in plain English with diagnosis and recommendations up front. Advice specific to the whole family, including siblings.

Parents also said that support in terms of talking to their child's school about strategies to put in place, as well as ones to use at home, would have been useful. Also, a further appointment would be valuable after diagnosis, once families have had time to read and digest the assessment report.

## 4 Discussion

This study provides a detailed analysis of parents' experiences while waiting for an ADHD assessment and diagnosis, and the impact this had on them and their child. Using parents' own narratives to explore current challenges in ADHD provisions and areas where the services could be improved, it provides new insight into the complexities of the waitlist experience.

Parents revealed that the period of waiting, from the point of being accepted into the service and the subsequent assessment, had a substantial psychological and practical impact on them and their child. This time was associated with many challenges, which are captured in detail in the six themes presented in this paper. Challenges concerned getting timely support for their child, child and parent mental health needs and educational needs. Parents also shared the type of support that could be useful to them in the assessment and diagnostic process, as well as suggestions for aftercare.

Parents acknowledged that they found support from the school lacking before a diagnosis had been established. With demands on CAMHS limiting the resources available to support education, it may be that schools need to do more to help children waiting for an ADHD diagnosis and make suitable adaptations, even if a diagnosis has not been established. Some local authorities have taken the initial steps to improve their services for families in need of psychological support. For example, Portsmouth City Council recently piloted the Neurodiversity (ND) profiling tool, which replaces the previous referral process where parents were waiting 18 months or more for an assessment [23]. The ND tool offers early profiling from trained professionals, giving parents and educational settings effective tools to manage behaviour, even without a diagnosis, meaning that children with ADHD type symptoms could receive early intervention while waiting for an assessment/diagnosis. Moreover, a study exploring the efficacy of Senior Mental Health Leads (SMHL) in schools found that SMHL were able to offer immediate support and reduce stigma around mental health through education [24]. However, the study also acknowledged the importance of the specialist knowledge offered by CAMHS, meaning an integrated approach is key.

The second key area concerns the need to improve how services manage waitlists and communication with those who use them. Parents communicated concerns around wait time, requesting transparency and clarity from services. Lengthy wait times have been found to be a barrier to young people accessing mental health support [8, 25]; however, there is a paucity of research on how services manage wait times. Implementing a quality improvement project allowed one trust to significantly reduce wait times for parents; modifications included changes to appointment letters stipulating the importance of attendance, thus reducing wasted appointments and having one clinician coordinating care rather than two [26]. Furthermore, a partial booking system for an eating disorder clinic gave patients more autonomy over their appointments, reducing non‐attendance and freeing up appointments for other patients [27]. With ADHD assessments generally taking a minimum of two appointments, parents may find an online booking system helpful, rather than waiting to be offered an appointment via letter. Offering non‐traditional locations and times may also go some way to reduce lengthy wait lists [13].

Parents offered insight into how services could be improved. Importantly, they wished to see a system implemented whereby they were regularly contacted (and thus reassured) by services, which included receiving more support and guidance while waiting. This echoes a study on parents and adolescents' experiences of CAMHS, which found that parents wanted more information on procedures prior to appointments as well as more behavioural support while they were waiting [28]. My HealthE, an online portal that allows families to access their local CAMHS online, has been designed to give personalised support, such as local support organisations, as well as information on what to expect during appointments, something that our study highlighted was lacking in many services [29]. A usability study highlighted some barriers to digital platforms such as the need for information to be tailor made and concerns about the lack of human contact; however, seeing their data summarised and being able to access information for their child was seen as benefits of the myHealthE platform [30].

The third area concerned support for parents themselves. Parents spoke of the impact that the lack of support had on them and their child while they waited for an assessment and diagnosis. Offering psychoeducation to help parents manage their child's behaviour during this time would be useful. A systematic review exploring the efficacy of WLIs, which included elements of psychoeducation, found improvements in child behaviour and parental anxiety [14]. However, in a survey of current waitlist provisions with CAMHS, Valentine et al. found that staff resources and funding were barriers to offering WLIs [15]. More research around cost effectiveness, as well as the efficacy of self‐guided digital support to reduce pressure on staff, would be useful.

### 4.1 Strengths and Limitations

With limited research on parents' experiences of waiting for an ADHD assessment/diagnosis with CAMHS, our paper adds valuable knowledge which can help shape and improve current systems and inform policy changes. PPI members working on the study were able to identify with many of the emerging themes, having shared similar experiences to the participants. This was reflected throughout the analytical process, which allowed for a deeper understanding of the data.

While we aimed to include a diverse range of participants from different ethnic and socioeconomic backgrounds, 63% of the participating parents were well‐educated, and the majority were female, with only two fathers taking part. Future research should aim to incorporate fathers' experiences for a more balanced perspective.

### 4.2 Implications for Changes in Practice

With lengthy wait times affecting children both at home and at school, mental health services need to adapt the way in which services operate. The UK government recognises the problem, and the latest report from the Government ADHD taskforce published in November 2025 makes 15 recommendations for change, including better support while waiting (providing clear information about wait times as well as offering help to those waiting) and making changes to how services work to speed up the process [3].

Several systems already exist which would merit further research on efficacy and scalability; online systems such as myHealthE [29] provide an online resource for parents without the need to contact services. Placing resources online allows parents to access signposting and support without having to wait for services to send information out/give access to information, reducing pressure on staff. Digitalising booking systems may reduce wasted appointments and give parents a greater sense of agency. An ND profiling tool, such as that piloted in Portsmouth City Council, provides early support for children, even before they receive a diagnosis, and reduces the lengthy wait times that are a major concern for parents.

## 5 Conclusion

Our study highlights the concerns and barriers faced by parents when waiting for an ADHD assessment/diagnosis for their child, specifically in the ways in which parents receive information on wait times, appointments and signposting from services. Increasing the efficacy of CAMHS without compromising its service and support of children and young people will be key in ensuring families receive the support needed. Many tools and platforms already exist or could be developed to meet the needs of CAMHS; this would not only give parents more autonomy in the way that they manage their time on a wait list and how they access much‐needed information, but also alleviate the burden on mental health services, resulting in a more efficient service.

## Author Contributions

**Ellen Hedstrom:** writing – original draft, participant interviews, transcription, qualitative data analysis, writing – review and editing. **Katarzyna Kostyrka‐Allchorne:** conceptualisation, writing – original draft, supervision, project administration, writing – review and editing. **Claire Ballard:** participant interviews, transcription, qualitative data analysis, writing – review and editing. **Naomi James:** transcription, qualitative data analysis, writing – review and editing. **Hannah Wright:** participant interviews, transcription, qualitative data analysis, writing – review and editing. **David Daley:** writing – review and editing, writing – review and editing. **Cris Glazebrook:** writing – review and editing. **Jana Kreppner:** writing – review and editing. **Claire Cattel:** interview schedule shaping, qualitative data analysis, writing – review and editing. **Douglas Gordon:** interview schedule shaping, qualitative data analysis, writing – review and editing. **Natalie Gordon:** interview schedule shaping, qualitative data analysis, writing – review and editing. **Tessa Tuttlebee:** interview schedule shaping, qualitative data analysis, writing – review and editing. **Edmund Sonuga‐Barke:** conceptualisation, methodology, data curation, formal analysis, overall OPTIMA Chief Investigator, writing – original draft, writing – review and editing.

## Disclosure

The views expressed are those of the authors and not necessarily those of the NIHR or the Department of Health and Social Care. The funding body played no role in the study design and conduct.

## Ethics Statement

Ethical approval for the trial was obtained from IRAS ID: 303121. Name of REC: Liverpool Central. REC reference number: 21/NW/0319. Date of favourable ethical opinion: 21 November 2021.

## Consent

Informed consent was given by parents who took part in the interviews via an electronic consent form signed and dated by the parent. At the point of consent into the trial, parents were asked to tick an optional question, *‘I agree to take part in the extra interviews and for the interviews to be audio/video recorded. I understand the video recording will be transcribed using Microsoft Stream and/or a member of the research team. I agree for anonymised quotes to be used for publication purposes’.* Parents who had consented to take part in interviews via the consent form were sent a participant information sheet explaining the study. At the point of the interview, parents were again asked to verbally consent that they were happy for the interview to commence.

## Conflicts of Interest

The authors declare no conflicts of interest.

## Supporting information

AppendixA_TopicGuide.

## Data Availability

The data that support the findings of this study are available on request from the corresponding author. The data are not publicly available due to privacy or ethical restrictions.
