# Supplementary material for: ‘Until You Get the Diagnosis You're Forever in Limbo’—Parents' Experiences of Waiting for an Attention‐Deficit/Hyperactivity Disorder Assessment With Child and Adolescent Mental Health Services
Source: Health Expect. 2026 Jan 28;29(1):e70569. doi: 10.1111/hex.70569 (PMC12848897; doi:10.1111/hex.70569)
Supplement: Supplementary file 1 — AppendixA_TopicGuide. [file HEX-29-e70569-s001.docx]

**Appendix A - Topic Guide**

Before the interview, the researcher should double check the consent form to ensure the parent has consented to being contacted for interviews.

Explain that:

- All parents who have taken part in the OPTIMA trial up to the final timepoint (12 months) and who have consented to be contacted for interviews have been invited to talk about their experiences of waiting for an assessment and diagnosis with (CAMHS/Notts/GHC).
- Explain that the team are collating the of experiences of families on the waiting lists.
- The interview will take around 30-45 minutes.
- You do not have to answer any questions you are not comfortable with and there are no right or wrong answers.
- You can take a break at any time or stop the interview, no explanation needed.
- If a question doesn’t make sense, ask for an explanation.
- You will receive a £20 Amazon voucher as a thank you for taking part. A voucher will be emailed to you within the next 5-7 days
- The original transcription and audio recording will be stored in a restricted-access folder on the King’s College London (KCL) cloud server, and no one other than immediate members of the research team can access this.
- Anonymised quotes from transcripts will be used in written reports, published journal articles and presentations including online. Again, any reference to places/family members/clinicians and so on will be removed. Pseudonyms may be used.

*Ask*: Do you have any questions about how we use your comments? Please feel free to ask anything however minor it may seem at this stage or at any time later.

*Ask*: Is it okay to record the interview?

*Ask*: Do you have any questions before we start?

*Ask*: Is it okay for me to start recording now?

*State researcher’s name, date, time, and OPTIMA ID (for data management)*

**Interview start**

Remind them of the date they entered the study, for example “you enrolled in the OPTIMA study on the [date] and it is now the [date] and we would like to find out a bit more about how the past year has been in terms of [child’s name] assessment and diagnosis.”

**Referral and assessment**

- Firstly, can you tell me a bit about how you got referred to [name of service]? Can you tell me whether you had any expectations at the time of being accepted onto the waitlist? What were your initial feelings about this?
- Can you tell me a bit about what has happened in the past year with your child’s journey? Prompt: Did you have an assessment during that time? Do you remember the date of the assessment? Was this with the services you were originally referred to? (allow to mention private if needed).
- Can you tell me a bit more whether you knew what was happening during the assessment and what they were testing for? What was it like for you? What was it like for your child?
- Can you tell me about whether you knew what to do if your child’s behaviour or mental health got worse during this time? Can you tell me about whether you were told anything about crisis services?
- How was information about the expected wait period communicated? Prompt: By letter, email? Did you feel that you were given information about how long you would need to wait? Were you given any updates about changes to wait times?
- Can you tell me about whether you received any support while waiting? [Explain that this could be very general e.g., flyers, links to websites, or more personal, such as phone calls from the staff]. If you did receive any support, what sort of support did you receive and was it helpful? If you didn’t receive anything, what support would you like to have received.
- Can you tell me about whether you looked for any additional support yourself (e.g., online resources, face-to-face or online parent groups, family or friends)? Can you tell me about the kind of things that you found helpful?
- What kind of support would you like to receive during wait time? What would help you and your family during this time?

**The child**

Is your child aware of the referral? If yes, how do they feel about it. Has your child talked about the assessment during the waiting period? How have they felt about it? Have you noticed any changes in your child during this period? Prompt: Mood, behaviour, school.

**Diagnosis**

- Has your child received a diagnosis yet? If yes, what was the diagnosis for and how did you feel on receiving a formal diagnosis for your child? If no, had you expected a diagnosis at this stage? How do you feel about the length of time it is taking?
- Can you tell me whether your child has been offered any treatment? Prompt: Pharmacological or psychological. How did you feel about your child being offered/not offered treatment?

**After care**

- After you received the diagnosis, how did you feel about the information or support you were given? Prompt: Did you receive any information leaflets for example? Did you receive appointments for further meetings or therapy? How were you signposted?
- Can you give me your thoughts or feelings on the process so far. Prompt: How has it made you or your child feel? If you could change it, what would you change?
- Do you have any advice for parents who have just been accepted onto the waiting list? Could you have done anything differently?

**Interview end**

That is the end of our interview. Do you have anything else you wish to tell me that I haven’t covered?

I am going to stop recording now. Thank you for your time.

*Stop recording.*

**Mood repair:** Ask if they feel OK. Offer to send out safety card again if needed. Ask if they have any nice plans for later in the day. Say experiences like these are not unusual. Say that the team cares and wants to bring difficulties/struggles to light and will use this research to try to do so.
